# Early impact of a new food store intervention on health-related outcomes

**DOI:** 10.1186/s12889-024-19052-1

**Published:** 2024-06-24

**Authors:** A. M. Hasanthi Abeykoon, Suvadra Datta Gupta, Rachel Engler-Stringer, Nazeem Muhajarine

**Affiliations:** 1 Department of Community Health and Epidemiology, University of Saskatchewan, Health Science Building, 107 Wiggins Road, Saskatoon, SK S7N-5E5 Canada; 2 Present Address: Saskatchewan Population Health and Evaluation Research Unit, Saskatoon, SK Canada; 3 University of Melbourne, Parkville, Victoria, Australia

**Keywords:** Population health intervention, Household food insecurity, Health-related outcomes, Moderator, Dose-response association

## Abstract

This study investigated the early impact of a community-based food intervention, the Good Food Junction (GFJ), a full-service grocery store (September 2012 – January 2016) in a former food desert in Saskatoon, Canada. The hypothesis tested was that frequent shopping at the GFJ improved food security and selected health-related outcomes among shoppers, and the impact was moderated by socioeconomic factors. Longitudinal data were collected from 156 GFJ shoppers, on three occasions: 12-, 18-, and 24-months post-opening. Participants were grouped into three categories based on the frequency of shopping at the GFJ: low, moderate, and high. A generalized estimating equations approach was used for model building; moderating effects were tested. Participants were predominantly female, Indigenous, low-income, and had high school or some post-secondary education. The GFJ use was associated with household food security (OR for high and moderate frequency shoppers reporting less than a high school education were 1.81 and 1.06, respectively), and mental health (OR for high and moderate frequency shoppers reporting high income were 2.82 and 0.87, respectively) exhibiting a dose-response relationship, and indicated that these outcomes were significantly moderated by participants’ socioeconomic factors. Shopping at the GFJ had a positive effect on food security and mental health, but to varying levels for those with low incomes, with less than high school or high school or better levels of education.

**Supplementary Information:**

The online version contains supplementary material available at 10.1186/s12889-024-19052-1.

## Introduction

Low-income neighborhoods frequently face challenges in ensuring equitable access to affordable and healthy food options for their residents. Often, these communities tend to have a higher concentration of fast-food or convenience stores compared to fewer grocery stores, which usually offer the most extensive range of healthy and affordable food [1–3]. Saskatoon, a mid-sized city in the province of Saskatchewan, Canada, has seen a sharp decline in the number of grocery stores since 1986, especially in the low-income neighborhoods, also known as the city’s ‘core neighbourhoods’. By 2004, Saskatoon’s lowest-income neighborhoods had no major chain grocery stores but had a high concentration of fast food and convenience stores. These neighbourhoods are identified as primary food deserts in Saskatoon with high poverty rates, higher unemployment rates, houses in need of major repair, single-parent families, and a greater number of residents of Indigenous ancestry. Residents living in these core neighbourhoods also have unequal health outcomes, with higher rates of chronic diseases such as diabetes, heart disease, psychological conditions, and all-cause mortality when compared to more affluent neighbourhoods in Saskatoon [4, 5]. A lack of grocery stores in the core neighbourhoods, therefore, meant disparities in accessing nutrient-rich food for equity-seeking groups, where a significant proportion of people, without a car, needed to spend up to an hour to reach a grocery store.

The Good Food Junction (GFJ), a unique food environment intervention of a full-service, not-for-profit cooperative grocery store with a community-led business model, opened in September 2012, presenting a much-needed intervention for core neighborhoods as identified in previous studies [6, 7]. Lifetime membership of this cooperative store was 5 Canadian dollars, and this fee was waived for many members [8]. The GFJ was one service component of a larger community services and enterprise hub, Station 20 West, facilitating access to services such as housing, food access, community outreach, and engagement. A survey was conducted within a year of GFJ’s opening among residents of four neighborhoods living within a 10-minute walking distance of the store. The study found that an increased number of Indigenous residents with low incomes used the store along with food-based programs, while recent immigrants used it significantly less. The majority of the residents (95%) were aware of the store’s existence, and 69% had used it at least once. However, only 8.2% of households had shifted to GFJ as their primary grocery store after the first 12 months of opening [3]. Another study investigated sales data of GFJ members for a year, starting eight months after its establishment [8]. This study revealed that residents living in core neighborhoods spent more on vegetables and less on meat and prepared foods compared to those living outside of the core neighborhoods. These results suggest that the core neighborhood residents were aware of GFJ and used it, and that this intervention was reaching the populations for whom it was intended. However, the impact of GFJ on health-related outcomes remains unanswered, which is the focus of the present study.

To date, a limited number of studies have investigated the impact of introducing new food store interventions in deprived, urban regions with low grocery store access. Inferring causation based on available evidence to guide programs and policies for new food store interventions is challenging due to a multitude of factors; consumer demand being one of them [9]. A systematic review of small food store interventions, a relevant context for our study, revealed significant improvements in sales of healthy foods and improved dietary behavior [10]. A study assessing the impact of hypermarket interventions found slight improvements in fruit and vegetable consumption but substantial improvements in psychological health [11]. However, there were a dearth of published reports examining the impact of a full-service grocery store intervention on health outcomes that were offered as a non-profit model. We aim, therefore, to examine if those who shopped in the GFJ more frequently over a 12-month period had better health-related outcomes compared to those who shopped less frequently.

## Methods

### Study design

A longitudinal survey design was employed on a cohort of regular shoppers of the GFJ over a 12-month period. The study surveyed participants 10–13 months (July-September 2013) after opening the GFJ (termed Round 1) and were contacted again on two occasions at 17–19 (February-April, 2014) (termed Round 2), and 23–25 (August-November, 2014) (termed Round 3) months. The aim was to space follow-up rounds by at least 6 months.

### Recruitment

Respondents who were the primary food shoppers for their household and who had shopped at least three times at the GFJ during the previous two months were initially recruited. Participant recruitment was done in four ways; (1) research assistants approaching shoppers in the store, at various times and on different days over a two-week period, (2) identification of further participants referred by already recruited participants, (3) distribution of flyers throughout the neighborhood near the store, and (4) further recruitment during the GFJ one-year anniversary celebration. Informed consent was obtained from all the participants before commencing the study. Ethical approval for this study was obtained from Behavioural Research Ethics Board of the University of Saskatchewan. Some of these methods have been published previously [12].

### Data collection and the survey

The paper survey was administered either in person at the store or if a participant preferred later over the telephone or in person. The survey, which was administered during three rounds, consisted of two components, and is provided in the supplementary material. The first component was regarding participants’ demographics and the second component focused on their health status. Both survey components were primarily consisted of several modules of 2011 version of the Canadian Community Health Survey (CCHS) [13], and were pre-tested and modified prior to using in this study.

The demographic part of the survey included questions on household socio-demographics such as age, sex, Indigenous status, highest education attained and annual household income. Specific questions relating to the GFJ including, how often they shopped at the GFJ and whether the GFJ was their primary grocery store were also included in this part of the survey.

The health status part of the survey asked questions on self-reported physical and mental health, changes made to improve health, chronic conditions, household food insecurity (HFI), and perceptions of neighbourhood residence. Since previous studies have found that a greater sense of social connectedness to the neighbourhood in which one lives is associated with better health outcomes [14], using 4 questions on the sense of belonging to the neighbourhoods, we derived an indicator of social connectedness to the neighbourhood.

### Measures

#### Dependent variables: HFI status and self-reported general health and mental health

The 18-item Household Food Security Survey Module (HFSSM) from the CCHS was used to assess HFI in this study [15]. It assesses HFI over the past 12 months for adults and children living in the household and reports the subjective experience of the participant on food insecurity, including anxiety and perceptions of household food supply and food intake [16]. The HFI status was determined by assessing the number of affirmative answers to the 18-item HFI module. Outcome categories were food secure (0–1), moderate food insecure [2–5], and severely food insecure (≥ 6) for adult and food secure (0–1), moderate food insecure [2–4], and severely food insecure (≥ 5) for children part of questions. It was hypothesized to be changed due to exposure to the GFJ intervention, and, in turn, a factor that plays a role midway between the exposure and general health-related outcomes.

Self-reported general health and mental health were also assessed based on questions obtained from the CCHS and asks about participants’ general health and mental health according to their perception on a 5-level scale (excellent, very good, good, fair, and poor).

### Key exposure of interest

Exposure to the GFJ intervention was captured using the item ‘how often have you shopped at the GFJ since it opened’. Based on the distribution of responses to this question a new variable— ‘dose’ or ‘frequency’ of exposure, was created with three levels: low = less than once a month since GFJ opened, moderate = about once a month since GFJ opened, and high = more than once a month since it opened. Dose of exposure to the GFJ for a given participant at a given data collection round was used as reported in that data collection round in the analysis, so that a participant could have different doses in different data collection rounds. Food purchasing patterns of GFJ shoppers have previously shown that they spent more food dollars on healthy food and less on prepared foods [8], supporting that high GFJ exposure (more frequent shopping) leads to better food choices.

### Co-variables

Health-related outcomes that were examined among GFJ users may be influenced by other factors in addition to the primary exposure of interest. Four types of such risk factors were identified:

#### Sociodemographic risk factors

age (senior citizen who are 65 year or older), sex, ethnicity (Indigenous vs. non-Indigenous), annual household income (low—$30,000 or less/ high—more than $30,000), participant’s education (less than high school/ high school and some post-secondary/ university).

#### Pre-existing health conditions and related risk factors

ever/ never diagnosed by a medical provider for diabetes, high blood pressure, heart disease, or cancer. Other health behaviour-related risk factors included level of daily stress, level of daily physical activity, and beliefs in changing health behaviour which included motivation and willingness to improve own physical health)

#### Perceived neighbourhood sense of belonging

We used categorical principal component analysis (CATPCA) to derive a single neighbourhood sense of belonging indicator based on four questions on neighbourliness as they were highly correlated.

#### Other

Other variables included choosing GFJ as the primary grocery store, duration of living in the neighbourhood, and the use of other community-based food programs.

### Analysis

A generalized estimating equations (GEE) approach with exchangeable working correlation structure was used for modelling. This equation efficiently estimates regression parameters of longitudinal (therefore correlated and not independent, but independent across individual participants) data using a quasi-likelihood function, and considers within-subject correlation as a ‘nuisance’ variable [17, 18].

Standard model building strategies were followed. Briefly, a univariate analysis selected variables with *p* < 0.25 which were retained for the multivariate model. The multivariate model selected variables with *p* < 0.05 which were retained for the preliminary final model [19]. The main predictor (GFJ exposure) was retained regardless of its level of significance. The preliminary final model was then subjected to assessment of two-way interactions between (i) the longitudinal ‘time’ and other covariables and (ii) the main predictor and other covariables tested one at a time. Interactions significant at *p* < 0.05 were retained for the final model and marginal (binary/categorical form) probability estimates were computed [19]. Model fit was determined using Quasi-Likelihood under Independence Model Criterion (QIC) and Corrected Quasi-Likelihood under Independence Model Criterion (QICC) values. The smaller QIC and QICC values show ‘better’ model fit, and the final models were adjusted accordingly [20]. Estimation of specific odds ratios in the presence of interaction was initially calculated manually [19]. SPSS (version 23, IBM) was used for all analyses. SAS 9.4 was used to confirm the odds ratios of the interaction terms, which were calculated by hand.

## Results

**Study sample**: The study initially enrolled 156 participants who completed the survey in Round 1. By Round 2 and 3, there were 27 and 37 participants lost to follow-up, respectively. Reasons were death (*n* = 2), refused further participation (*n* = 1) and unable to establish contact using the contact information provided. The sample was replenished by recruiting 24 new participants during Round 2, which restored the composition of the cohort (sociodemographically) similar to Round 1. This resulted in 153 participants completing Round 2 and 115 of them completed Round 3. All three rounds were completed by 104 while 37 participants completed only two rounds. A comparison between study completers and non-completers along the sociodemographic, independent and dependent variables showed that the participants who were food insecure, Indigenous, had less than high school education, low-income, and who had lived in their neighbourhood less than 5 years were statistically more likely to not complete all three follow-ups. The details are provided in the supplementary material (Table S1).

**Characteristics of participants recruited during round 1 and round 2**: The majority of study participants, across the three data collection rounds, were female, about one-half self-identified as Indigenous, had low-incomes, and had at least a high school or some post-secondary education. Table 1 shows characteristics of participants.

**Table 1:** Participant characteristics during three rounds of data collection

Characteristics	Round 1	Round 2	Round 3
Number of participants	156	153	115
Age [median (min, max)]	42 (21, 90)	43 (21, 91)	44 (22, 91)
Sex [n (%)] ♣ Male ♣ Female	39 (25) 117 (75)	38 (24.8) 115 (75.2)	29 (25.2) 86 (74.8)
Self-identified Ethnicity [n (%)] ♣ First Nations Status ♣ First Nations Non-Status ♣ Métis ♣ Inuit ♣ Total Indigenous (% of total sample)	53 (32.5) 8 (4.9) 19 (11.7) - 79 (50.6)	60 (38.2) 3 (1.9) 19 (12.1) - 81 (52.9)	34 (28.1) 2 (1.7) 12 (9.9) - 46 (40.4)
Newcomers to Canada (< 5 years in Canada) [n (%)]	4 (2.5)	-	1 (0.8)
Annual household income [n (%)] ♣ Less than $30,000 ♣ $30,000 or more ♣ Don’t know or decline to answer	85 (54.5) 49 (31.4) 22 (14.1)	93 (60.8) 44 (28.8) 16 (10.5)	69 (60.0) 38 (33.0) 8 (7.0)
Highest level of education [n (%)] ♣ Less than high school ♣ High school & some post-secondary/ technical college ♣ Completed university	45 (28.8) 77 (49.4) 34 (21.8)	43 (28.1) 78 (50.9) 32 (20.9)	22 (19.1) 61 (53) 32 (27.8)

The level of GFJ exposure increased positively except high exposure in Round 3. As well, HFI followed a positive trend with a gradually increasing food security (from 45.5 to 63.5%) and concomitant falling food insecurity (from 54.5 to 36.5%) over three follow-up Rounds. Other self-reported health-related outcomes fluctuated over the follow-up period without a particular pattern. These results are presented in supplementary material (Table S2).

**Health-related outcomes**: Tables 2, 3 and 4 present summarized odds ratios (ORs), their 95% confidence intervals (CIs) and significance levels in univariate and multivariate GEE analyses. The final model showed that Indigenous ethnicity and senior age no longer significantly contributed to predicting HFI in this sample of GFJ shoppers (Table 2). The level of education significantly modified the effect of GFJ exposure in predicting HFI.

**Fig. 1:**
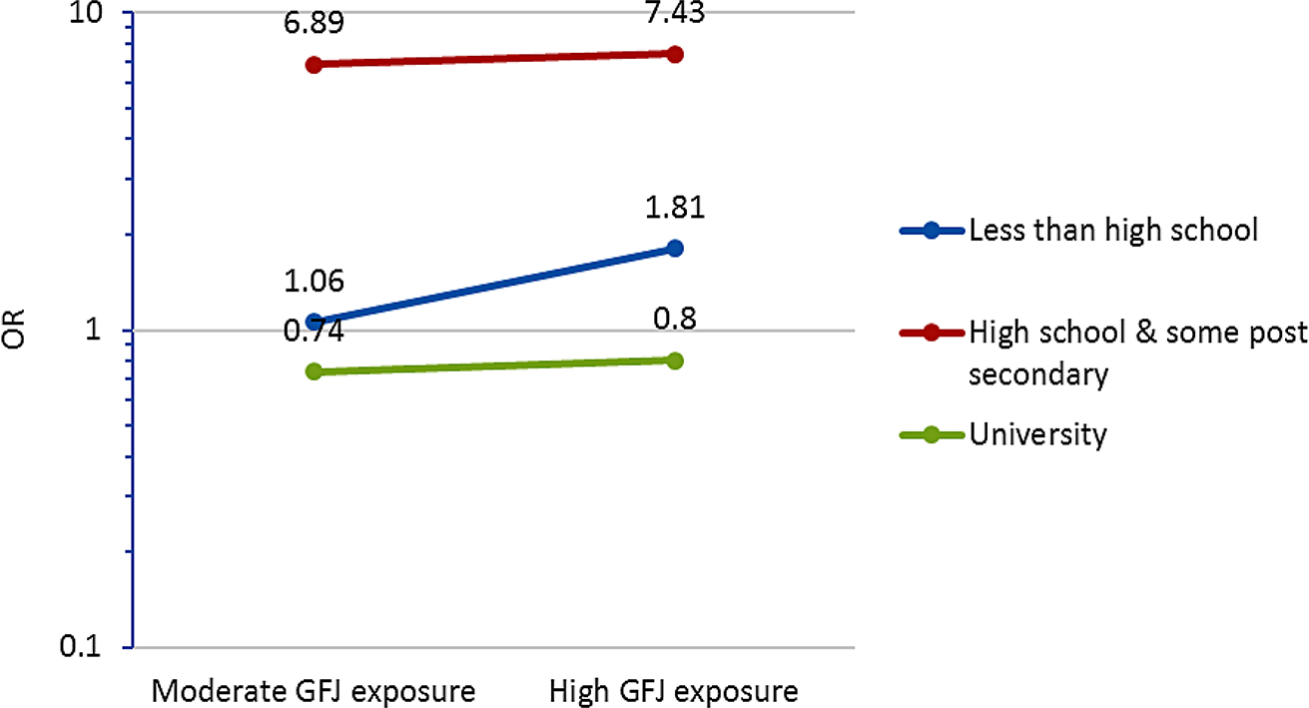
Frequency of shopping at the GFJ and odds ratio of household food security by the level of education

A ‘dose’-dependent association between the frequency of GFJ use and the odds of reporting food security was found, and this association was significantly modified by participants’ education level. The positive impact on food security with GFJ exposure was greatest among participants with less than high school education. Figure 1 illustrates how OR of the main association of GFJ exposure and food security is modified by the level of education. The likelihood of reporting food security among GFJ shoppers with high school or some post-secondary education was notably high for those who shopped frequently (high) or moderately frequently compared to those who shopped less frequently (OR = 7.43 CI 1.81, 30.44, *p* = 0.005; OR = 6.89 CI 1.57, 30.20, *p* = 0.010). In Fig. 1 this is shown by the highest location (red line) on the y axis. As shown in Fig. 1, the likelihood of reporting food security increased slightly among high frequency and moderate frequency GFJ shoppers (OR = 1.81 CI 0.42, 7.74, *p* = 0.425; and OR = 1.06 CI 0.17, 6.48, *p* = 0.948) for those who reported less than a high school education. In contrast, the impact on food security associated with shopping at GFJ was the least among those who had university level education. Further, there is a marked difference in slopes of the lines within each education level in Fig. 1. For instance, the blue line has a steeper slope indicating that the GFJ shoppers with less than high school education showed higher odds of food security with higher exposure implying greater effect with increasing dose.

Participants with low income (less than $30,000 household income per year) were approximately 76% less likely to be food secure (lower odds) over three-time points compared to participants with higher incomes. Further, male participants were approximately 2.32 times (95% CI 1.16, 4.66; *p* = 0.018) more likely to be food secure over three-time points compared to female participants. Participants with high and moderate level of connectedness to the neighbourhoods they lived in were 2.04 times (CI 1.09, 3.83; *p* = 0.027) and 1.33 times (CI 0.75, 2.37; *p* = 0.331) more likely to be food secure, respectively, over three-time points compared to participants with low neighbourhood connectedness. Moreover, those who used 3 or more other food-based programs were approximately 65% less likely (OR = 0.35 CI 0.13, 0.96; *p* = 0.041) to be food secure, while those who used only 1 or 2 of those programs were 73% less likely (OR = 0.27 CI 0.09, 0.79; *p* = 0.017) to be food secure compared to participants who did not use any of the other food-based programs.

**Table 2:** Univariate and multivariate models showing factors independently associated with household food security

Household food security status: 1 = food secure| indicator 0 = moderate & severe food insecure| reference		Univariate		Multivariate	
Variable	Reference category	Odds ratio (95% CI)	*p*-value	Odds ratio (95% CI)	*p*-value
Exposure level to GFJ High Moderate	low	1.65 (0.98, 2.79) 1.74 (0.97, 3.12)	0.060 0.063	0.80 (0.31, 2.03) 0.74 (0.30, 1.84)	0.634 0.520
Senior	Not senior	2.44 (1.33, 4.5)	0.004		
Low income	High	0.18 (0.09, 0.34)	0.000	**0.24 (0.12, 0.50)**	**0.000**
Education Less than high school High school & some post secondary	university	0.16 (0.07, 0.35) 0.22 (0.11, 0.42)	0.000 0.000	0.13 (0.02, 0.70) 0.04 (0.01, 0.19)	0.017 0.000
Male	Female	1.97 (1.09, 3.59)	0.026	**2.32 (1.16, 4.66)**	**0.018**
Indigenous identity	Non- Indigenous	0.35 (0.21, 0.58)	0.000		
Daily stress	Not stressful	0.88 (0.65, 1.19)	0.414		
Physical activity	Low	0.89 (0.64, 1.24)	0.493		
Pre-existing chronic conditions	Never	1.25 (0.79, 1.98)	0.347		
Believe in changing health behaviour	Low	0.93 (0.69, 1.26)	0.621		
How long lived in the neighbourhood	< 5 years	1.16 (0.80, 1.69)	0.427		
Neighbourhood connectedness High Moderate	Low	1.79 (1.12, 2.84) 1.15 (0.76, 1.75)	0.016 0.500	**2.04 (1.09, 3.83)** 1.33 (0.75, 2.37)	**0.027** 0.331
GFJ primary grocery store	No	0.98 (0.68, 1.42)	0.917		
Use of other food-based programs 3 or more programs 1–2 programs	None	0.54 (0.27, 1.07) 0.44 (0.22, 0.86)	0.079 0.016	**0.35 (0.13, 0.96)** **0.27 (0.09, 0.79)**	**0.041** **0.017**
High GFJ exposure*less than high school				2.27(0.42, 12.19)	0.340
High GFJ exposure*high school & post secondary				**9.32 (1.76, 49.23)**	**0.009**
Moderate GFJ exposure*less than high school				1.43 (0.19, 10.55)	0.726
Moderate GFJ exposure*high school & post secondary				**9.28 (1.66, 51.69)**	**0.011**

*Note* See Fig. 1 where these interaction effects are graphed

The final model for self-reported health showed that participants who were low income and experiencing daily stress were 70% (*p* > 0.000) and 40% (*p* = 0.053) less likely to report good to excellent health over three-time points compared to participants who were high income and not experiencing stress daily, respectively (Table 3). Further, participants with less than high school education were 68% (*p* = 0.021) less likely to report good to excellent health over three-time points compared to participants with a university education. Participants ever having pre-existing chronic conditions were 63% (*p* = 0.002) less likely to report good to excellent health over three-time points compared to participants who never had chronic conditions.

**Table 3:** Univariate and multivariate models showing factors independently associated with good to excellent self-reported general health

Self-reported general health: 1 = good to excellent| indicator 0 = fair to poor| reference		Univariate		Multivariate	
Variable	Reference category	Odds ratio (95% CI)	*p*-value	Odds ratio (95% CI)	*p*-value
Exposure level to GFJ High Moderate	low	0.92 (0.55, 1.54) 0.99 (0.57, 1.72)	0.749 0.980	0.82 (0.43, 1.57) 0.86 (0.45, 1.64)	0.553 0.630
Senior	Not senior	0.63 (0.30, 1.35)	0.239	0.45 (0.18, 1.12)	0.086
Low income	High	0.32 (0.17, 0.60)	0.000	**0.30 (0.16, 0.57)**	**0.000**
Education Less than high school High school & some post secondary	University	0.01 (0.11, 0.67) 0.05 (0.21, 1.00)	0.005 0.047	**0.32 (0.12, 0.84)** 0.69 (0.30, 1.58)	**0.021** 0.373
Male	Female	1.28 (0.71, 2.31)	0.404		
Indigenous identity	Non- Indigenous	0.69 (0.37, 1.29)	0.246		
Daily stress	Not stressful	0.75 (0.50, 1.13)	0.170	**0.60 (0.35, 1.01)**	**0.053**
Physical activity	Low	1.03 (0.69, 1.54)	0.895		
Pre-existing chronic conditions	Never	0.40 (0.23, 0.69)	0.001	**0.37 (0.19, 0.69)**	**0.002**
Believe in changing health behaviour	Low	1.08 (0.77, 1.53)	0.646		
How long lived in the neighbourhood	< 5 years	0.85 (0.55, 1.31)	0.455		
Neighbourhood connectedness High Moderate	Low	1.56 (0.93, 2.61) 1.27 (0.82, 1.97)	0.092 0.293		
GFJ primary grocery store	No	0.71 (0.46, 1.10)	0.130		
Use of other food-based programs 3 or more programs 1–2 programs	None	1.13 (0.49, 2.57) 1.02 (0.50, 2.07)	0.776 0.967		

**Fig. 2:**
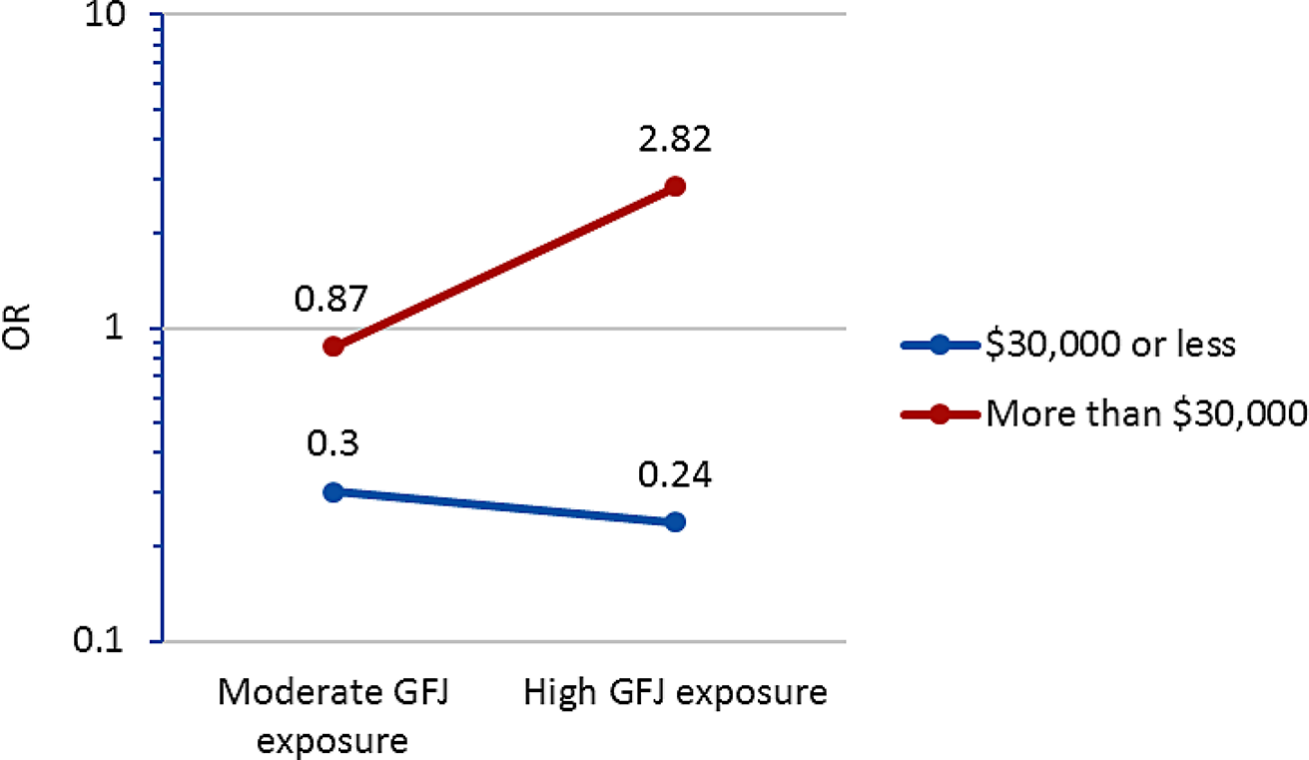
Frequency of shopping at the GFJ and odds ratio of self-rated excellent to good mental health by the level of income

As Table 4 shows, participants with daily stress were 68% (*p* = 0.001) less likely to report good to excellent mental health over the three time points compared to participants without stress. As shown in Fig. 2, the positive impact on mental health associated with shopping at the GFJ is notable for participants from relatively high income households as illustrated by the steep slope of the red line. Participants from households with income greater than $30,000 were 2.82 (95% CI 0.42, 18.93) times more likely to report good to excellent mental health if they shopped at high frequency at the GFJ, compared to less frequently. In fact, among those reporting the same level of income, those who shopped in ‘moderate’ frequency at GFJ were 13% (OR = 0.87; CI 0.25, 2.96) less likely to report good to excellent mental health. There were no benefits on mental health shown for those reporting income less than $30,000 regardless of frequency of shopping at GFJ (see Fig. 2 blue line).

**Table 4:** Univariate and multivariate models showing factors independently associated with self-reported mental health

Self-reported mental health: 1 = good to excellent| indicator 0 = fair to poor| reference		Univariate		Multivariate	
Variable	Reference category	Odds ratio (95% CI)	*p*-value	Odds ratio (95% CI)	*p*-value
Exposure level to GFJ High Moderate	low	0.65 (0.27, 1.53) 0.68 (0.32, 1.47)	0.319 0.325	2.83 (2.96, 97.71) 0.87 (0.25, 2.96)	0.284 0.819
Senior	Not senior	0.76 (0.28, 2.08)	0.589		
Low income	High	0.38 (0.14, 1.04)	0.060	1.87 (0.25, 14.28)	0.546
Education Less than high school High school & some post secondary	university	0.62 (0.21, 1.87) 1.06 (0.40, 2.83)	0.398 0.909		
Male	Female	1.38 (0.53, 2.63)	0.511		
Indigenous identity	Non- Indigenous	1.08 (0.53, 2.22)	0.835		
Daily stress	Not stressful	0.40 (0.21, 0.76)	0.005	**0.32 (0.16, 0.64)**	**0.001**
Physical activity	Low	1.12 (0.62, 2.04)	0.705		
Pre-existing chronic conditions	Never	0.66 (0.34, 1.27)	0.212		
Believe in changing health behaviour	Low	0.99 (0.54, 1.80)	0.963		
How long lived in the neighbourhood	< 5 years	1.30 (0.58, 2.93)	0.522		
Neighbourhood connectedness High Moderate	Low	1.23 (0.62, 2.48) 1.40 (0.77, 2.55)	0.554 0.273		
GFJ primary grocery store	No	1.10 (0.48, 2.51)	0.819		
Use of other food-based programs 3 or more programs 1–2 programs	None	0.83 (0.31, 2.22) 0.71 (0.28, 1.81)	0.706 0.478		
High GFJ exposure*low income				**0.08 (0.01, 0.83)**	**0.034**
Moderate GFJ exposure*low income				0.34 (0.05, 2.18)	0.256

Note See Fig. 2 where these interaction effects are graphed

## Discussion

The purpose of this study was to assess the early (within a year) health-related impact of a new grocery store intervention, the GFJ, in a former food desert. The study followed-up a sample of GFJ shoppers for one year and measured their HFI, self-reported general health, and mental health. Results showed positive impact of GFJ exposure among participants who shopped most frequently or moderately frequently compared to low. A few outcomes, however, did not corroborate the hypothesis.

Shoppers who shopped at the GFJ at least once a month since it opened (high and moderate frequency) were likely to report their health-related outcomes having improved compared to those who shopped less often than once a month. Participants with less than high school education were more likely to report food security over time when they shopped at the GFJ more frequently, and this improvement was notably heightened among participants who had high school or some post-secondary level education. Those shoppers with a university level education, however, were the least affected by the intervention. They showed better HFI status at the study start (data not shown), therefore, use of the GFJ at a moderate and high frequency improved HFI only very slightly. This cohort of participants may have had other options of healthy food sources that were not limited by the location or price, thus making them the group who least benefited from the GFJ intervention. It is an interesting and a useful finding that the shoppers with less than university level education (but at minimum a high school education) benefited the most from shopping at the GFJ in terms of HFI.

Similarly, those who shopped at the GFJ more often than once a month compared to those who shopped less than once a month showed a sharp rise in positive mental health among high-income participants. Low-income participants, however, had lower odds of positive mental health even when they shopped at the GFJ at a higher frequency. The implication of this result is that those who were exposed to this new food store intervention did not benefit equally in terms of improved health. The effect modification of the GFJ exposure on food security and mental health by education and income, respectively, suggest that this intervention was more effective among those who had less than a university education, but at minimum a high school education (regarding food security) or among those with high income (regarding mental health) [21].

The majority of shoppers (three-quarters of participants in each round) followed-up in this study did their primary grocery shopping at stores other than the GFJ. “High level of exposure” in this study referred to ‘more than once a month’ shopping at the GFJ, which describes a biweekly or weekly or more trips. Although grocery shopping frequency depends on age, socioeconomic status, household size and ethnicity, many studies show that the majority of households grocery shop at a frequency of more than once a month, particularly biweekly or weekly [22–27]. The bulk of the study participants doing their primary shopping for groceries at food stores other than the GFJ is something that we did not control in this study.

Household food insecurity measured nationally in 2021 using the CCHS indicates that 15.9% (representing 5.8 million people including 1.4 million children below 18 years) of Canadian households were food insecure (marginal, moderate or severe) during the previous year [28]. In Saskatchewan, HFI rose from 12.2% in 2013 to 18.8% in 2021 [28]. The participants in the current study showed a higher (54.5%) level of food insecurity (as expected) than the Saskatchewan or the national average at the start of the study which then reduced to 36.5% by Round 3. Although food insecurity improved in these GFJ shoppers over the three longitudinal time points, it was still considerably higher than Saskatchewan and national food insecurity levels.

The apparent improvement in food security in these study participants may be explained using four reasons. First, as this study hypothesized, the opening of the GFJ in the former food desert and use of this store by study participants might have led to an improvement in their HFI status for some participants.

Second, participants used other community-based food programs such as gardens, CHEP Good Food Boxes, Food Bank, Farmers’ Market, Collective kitchens, CHEP community markets, Seniors’ markets or other food programs. Statistical analysis indicates that participants who used multiple food programs were less likely to be food secure compared to those who did not use any of them. These community-based food programs are diverse in many respects and simply counting how many programs a participant had participated in without taking into account the specific nature of the program, or its effectiveness to enhance food security, is a limitation in this study. The present study identifies this limitation and proposes that future studies could take community-based food programs with regard to their nature into account.

Third, there is a possibility that the GFJ exposure may have also contributed to engagement with other health promoting services available through Station 20 West, which in turn could have contributed positively to food security. Further, a number of community-based programs (specifically CHEP volunteers, clients of KidsFirst program, immunization program, Healthy Mother Healthy Baby program) were giving coupons that could be redeemed for food at the GFJ and CHEP fresh food markets. So, it is possible that some people who frequently shopped at the GFJ may have done this because they had coupons that they could redeem at the GFJ. This could contribute to improvements in food security among frequent shoppers and function as increased income for food in the household which has been reported in the literature as contributing to improved HFI status [28].

Finally, the selective loss of study participants over three data collection waves and the change in the study sample due to new participant recruitment during Round 2 would have had an impact on food security and health-related outcomes changes. As evidenced by the significant differences between study completers and non-completers, participants who were the most food insecure were the ones that were lost to follow-up, resulting possibly in an overestimation of food security. However, countering this, participants recruited during Round 2 were significantly more food insecure compared to the cohort recruited at the study start.

National averages of perceived very good or excellent mental health in Canada (59.0% in 2021) are lower than that reported by this study (88.2–89.1%) [29]. As well, Saskatchewan averages (55.5% in 2021) are even lower than present study reports [29]. Overall GFJ shoppers reported slightly declining good to excellent mental health from first (89.1%) to third (88.7%) data collection waves. The present study found that establishing a grocery store in a former food desert improved HFI, and mental health of its users with time. Most importantly, participants who shopped at the new grocery store frequently were more likely to be food secure, and report better mental health than those who shopped at a moderate or low frequency (with these effects modified by a third variable). This ‘dose-response’ type association strengthen claims with regard to causation between the observed factors. Although graded relationships are not in the expected direction for all outcomes and general health did not show any significant improvements during the study period, the positive and dose-response association between food security and increasing levels of GFJ exposure might lead to improvements in other health-related outcomes later on. As expected, low-income and low-education were significant independent predictors of at least one of the outcomes studied—HFI, self-rated general health, and mental health, which is consistent with previous literature. The implication being that although physical access to food is improved, low socioeconomic status continues to be a major barrier to consuming healthy foods that are expensive and lower in caloric content than high sugar and high fat processed food [30]. Many similar previous studies included study participants who were only low-income or living in deprived neighbourhoods expecting higher positive impacts [11, 31–34]. Although the GFJ was also located in low-income neighbourhoods, participants of this study constituted GFJ shoppers from all over the city. Household income and the level of education of participants showed a fairly diverse distribution in this sample. This combination opened up an opportunity to compare different socioeconomic groups exposed to the GFJ intervention.

It has long been identified that individuals’ neighbourhood social ties play an important role in health [35, 36]. At the level of univariate analysis, this study found that perceived neighbourhood connectedness significantly (at *p* < 0.25 level) and in a dose-dependent manner predicted HFI and general health among this sample of GFJ shoppers. At the multivariate level, a higher level of neighbourhood connectedness showed higher odds of being food secure. Although this study did not find any significant moderating effect by neighbourhood connectedness on the outcomes assessed, the need to engage psychosocial moderators is rapidly being recognized in food environment research [37]. Future food environment interventions that accompany additional programs that engage the community and build up neighbourliness may, therefore, be more effective in promoting health than if these efforts were separated.

Although some core neighbourhood residents benefited from the new grocery store, the GFJ did not survive long. The store closed at the end of January 2016 due to low sales nearly 3.5 years after its opening. The study would have been strengthened if baseline data on this sample of GFJ shoppers were available to compare their health before opening the GFJ. The study sample from throughout Saskatoon, and not only from the surrounding neighbourhoods of the GFJ, makes the generalization of these findings difficult to similar inner-city low-income food deserts. This study did not evaluate the proportion of study participants living in the core neighbourhoods vs. the rest of Saskatoon. The neighbourhood of residence of participants and the transience of their residency through the study period might have had an impact on the frequency of shopping at the GFJ and the outcomes measured, which could be addressed in future research. Nevertheless, the geographical heterogeneity of residence of this sample was also a strength. The study participants represented a mix of socioeconomic status and Indigenous and non-Indigenous ethnicity, which contributed to factors such as income and education emerging as statistically significant predictors, as well as contributing to the generalizability of findings to other similar settings.

The method of participant recruitment might have introduced a risk of selection bias as it might have led the GFJ shoppers who were motivated to stay healthy to participate in the study. As well, participation in the study itself might have led to increasing awareness of healthy eating and other health-related behaviours among the participants, which might have contributed to changes over the longitudinal data collection waves. Another important limitation of this study is the selective loss to follow-up. There were significant differences between study completers and non-completers regarding sociodemographic risk factors, the main predictor as well as some of the outcomes measured. Participants who were lost over the three-time points were those who were the most food insecure. This might have created estimates that are biased towards more positive results.

In addition to being a natural, real-life experiment, this population health intervention brings much strength regardless of above-mentioned limitations. Participants reporting how often they shopped at the grocery store and using these data to create a ‘dose’ to assess the intervention ‘exposure’ is a key strength of this study. Prospective follow-up of study participants reduced any recall bias that may have arisen if retrospective methods were used. This key strength is intensified by the inferences derived using a GEE approach. GEE are based on marginal models and come up with population averages. Evidence produced from this study is therefore useful for population-level policy, practice, and program planning.

Having an integrated approach by controlling for most of the known covariables that determine health in addition to improved food access, namely individuals’ perceived neighbourhood connectedness, beliefs in changing health behaviour, socioeconomic status, senior age, Indigenous identity, daily experience of stress, physical activity, and pre-existing chronic conditions, provide a comprehensive picture. This type of analysis would be very useful for decision-making around future population health and targeted interventions.

## Conclusions

This food environment intervention study found that the opening of a grocery store in a former food desert improved the HFI and mental health of its users in a graded fashion. The establishment of the grocery store was originally a priority of core neighbourhood residents. The study shows that improving food security is only one aspect of a bigger problem of nutrition-related non-communicable diseases and health-related outcomes. There are many other factors at play which need careful planning at more upstream levels. For instance, low socioeconomic status continues to be a significant risk factor for health-related outcomes. Although reproduction of these findings in diverse contexts is highly recommended, a comprehensive approach in prevention program and planning strategies are emphasized.

### Electronic supplementary material

Below is the link to the electronic supplementary material.

Supplementary Material 1

Supplementary Material 2

## Data Availability

The datasets used and/or analysed during the current study are available from the corresponding author on reasonable request.

## References

[1] Hilmers A, Hilmers DC, Dave J (2012). Neighborhood disparities in access to healthy foods and their effects on environmental justice. Am J Public Health.

[2] Cushon J, Creighton T, Kershaw T, Marko J, Markham T. Deprivation and food access and balance in Saskatoon, Saskatchewan. Chronic Dis Inj Can. 2013;33(3). 23735454

[3] Lotoski LC, Engler-Stringer R, Muhajarine N (2015). Cross-sectional analysis of a community-based cooperative grocery store intervention in Saskatoon, Canada. Can J Public Health.

[4] Lemstra M, Neudorf C, Opondo J (2006). Health disparity by neighbourhood income. Can J Public Health.

[5] Saskatoon Health Region. Health disparity in Saskatoon: Analysis to intervention 2008 https://nccdh.ca/resources/entry/health-disparity-in-saskatoon.

[6] Woods F, Randall JE, Armstrong-Monahan C, Usiskin L, Whiting S, Waygood K, et al. Access to food in Saskatoon’s core neighborhood. Community-University Institute for Social Research; 2003.

[7] Engler-Stringer R, Harder J. Toward implementation of the Saskatoon Food Charter: a report. With the Saskatoon Food Coalition Community-University Institute for Social Research; 2011.

[8] Fuller D, Engler-Stringer R, Muhajarine N (2015). Examining food purchasing patterns from sales data at a full-service grocery store intervention in a former food desert. Prev Med Rep.

[9] Allcott H, Diamond R, Dubé J-P, Handbury J, Rahkovsky I, Schnell M (2019). Food deserts and the causes of nutritional inequality. Q J Econ.

[10] Gittelsohn J, Rowan M, Gadhoke P. Interventions in small food stores to change the food environment, improve diet, and reduce risk of chronic disease. Prev Chronic Dis. 2012;9. PMC3359101 22338599

[11] Cummins S, Findlay A, Higgins C, Petticrew M, Sparks L, Thomson H (2008). Reducing inequalities in health and diet: findings from a study on the impact of a food retail development. Environ Plan A.

[12] Engler-Stringer R, Muhajarine N, Ridalls T, Abonyi S, Vatanparast H, Whiting S (2016). The good Food Junction: a community-based food store intervention to address nutritional health inequities. JMIR Res Protocols.

[13] Government of Canada. Canadian Community Health Survey 2022 https://www.canada.ca/en/health-canada/services/food-nutrition/food-nutrition-surveillance/health-nutrition-surveys/canadian-community-health-survey-cchs.html.

[14] Muhajarine N, Vu LT (2009). Neighbourhood contexts and low birthweight: social disconnection heightens single parents risks in Saskatoon. Can J Public Health.

[15] Government of Canada. The Household Food Security Survey Module (HFSSM). 2012 https://www.canada.ca/en/health-canada/services/food-nutrition/food-nutrition-surveillance/health-nutrition-surveys/canadian-community-health-survey-cchs/household-food-insecurity-canada-overview/household-food-security-survey-module-hfssm-health-nutrition-surveys-health-canada.html.

[16] Jones AD, Ngure FM, Pelto G, Young SL (2013). What are we assessing when we measure food security? A compendium and review of current metrics. Adv Nutr.

[17] Liang K-Y, Zeger SL (1986). Longitudinal data analysis using generalized linear models. Biometrika.

[18] Twisk J. Continuous outcome variables-relationships with other variables. Applied longitudinal data analysis for epidemiology: a practical guide. 2003:77–88.

[19] Hosmer DW Jr, Lemeshow S, Sturdivant RX. Applied logistic regression: Wiley; 2013.

[20] Pan W (2001). Akaike’s information criterion in generalized estimating equations. Biometrics.

[21] Wu AD, Zumbo BD (2008). Understanding and using mediators and moderators. Soc Indic Res.

[22] Yoo S, Baranowski T, Missaghian M, Baranowski J, Cullen K, Fisher JO (2006). Food-purchasing patterns for home: a grocery store-intercept survey. Public Health Nutri.

[23] Jilcott SB, Moore JB, Wall-Bassett ED, Liu H, Saelens BE (2011). Association between travel times and food procurement practices among female supplemental nutrition assistance program participants in eastern North Carolina. J Nutr Educ Behav.

[24] Hirsch JA, Hillier A (2013). Exploring the role of the food environment on food shopping patterns in Philadelphia, PA, USA: a semiquantitative comparison of two matched neighborhood groups. Int J Environ Res Public Health.

[25] Liese AD, Bell BA, Barnes TL, Colabianchi N, Hibbert JD, Blake CE (2014). Environmental influences on fruit and vegetable intake: results from a path analytic model. Public Health Nutr.

[26] Kim B-D, Park K (1997). Studying patterns of consumer’s grocery shopping trip. J Retail.

[27] Wilde PE, Ranney CK (2000). The monthly food stamp cycle: shopping frequency and food intake decisions in an endogenous switching regression framework. Am J Agric Econ.

[28] Tarasuk V, Mitchell A, Dachner N. Household food insecurity in Canada, 2014. Toronto: Research to identify policy options to reduce food insecurity. Proof-Food Insecurity Policy Research–Toronto; 2016. 2020.

[29] Statistics Canada. Perceived mental health, by age group 2022 https://www150.statcan.gc.ca/t1/tbl1/en/tv.action?pid=1310009603.

[30] Handbury J, Rahkovsky I, Schnell M. What drives nutritional disparities? Retail Access and Food purchases across the socioeconomic spectrum. National Bureau of Economic Research Cambridge, MA; 2015.

[31] Dubowitz T, Ghosh-Dastidar M, Cohen DA, Beckman R, Steiner ED, Hunter GP (2015). Diet and perceptions change with supermarket introduction in a food desert, but not because of supermarket use. Health Aff.

[32] Elbel B, Moran A, Dixon LB, Kiszko K, Cantor J, Abrams C (2015). Assessment of a government-subsidized supermarket in a high-need area on household food availability and children’s dietary intakes. Public Health Nutr.

[33] Wang MC, MacLeod KE, Steadman C, Williams L, Bowie SL, Herd D (2007). Is the opening of a neighborhood full-service grocery store followed by a change in the food behavior of residents?. J Hunger Environ Nutr.

[34] Cummins S, Petticrew M, Sparks L, Findlay A. Large scale food retail interventions and diet. BMJ; 2005. pp. 683–4. 10.1136/bmj.330.7493.683 PMC555618 15790619

[35] Seeman TE (1996). Social ties and health: the benefits of social integration. Ann Epidemiol.

[36] Kawachi I, Berkman LF (2001). Social ties and mental health. J Urb Health.

[37] Mercille G, Richard L, Gauvin L, Kestens Y, Shatenstein B, Daniel M (2016). The food environment and diet quality of urban-dwelling older women and men: assessing the moderating role of diet knowledge. Can J Public Health.

