# Supplementary material for: Early impact of a new food store intervention on health-related outcomes
Source: BMC Public Health. 2024 Jun 24;24:1688. doi: 10.1186/s12889-024-19052-1 (PMC11197370; doi:10.1186/s12889-024-19052-1)
Supplement: Supplementary file 1 — Supplementary Material 1 [file 12889_2024_19052_MOESM1_ESM.docx]

**Supplementary files**

Loss to follow-up

**Table S1:** Characteristics of study completers and non-completers (based on round 1 data)

| **Characteristic** | **Study completers (%)** | **Non-completers (%)** | **Chi-square p-value** |
| --- | --- | --- | --- |
| GFJ exposure   - Low - Moderate - High | 19  16  65 | 27  16  57 | 0.508 |
| Food security   - Food secure - Food insecure | 53  47 | 32  68 | **0.012** |
| General health   - Fair to poor - Good to excellent | 23  77 | 34  66 | 0.140 |
| Mental health   - Fair to poor - Good to excellent | 12  88 | 9  91 | 0.555 |
| Use of other food programs   - None - 1-2 - 3 or more | 12  66  22 | 11  59  30 | 0.512 |
| GFJ primary store   - No - Yes | 76  24 | 73  27 | 0.700 |
| Aboriginal status   - No - Yes | 64  36 | 23  77 | **0.000** |
| Level of education   - University - High sch& some post sec - Less than high school | 31  49  20 | 5  50  45 | **0.000** |
| Household income   - High - Low | 43  57 | 23  77 | **0.020** |
| Length of time lived in neighbourhood   - Less than 5 years - 6 or more | 59  41 | 82  18 | **0.003** |

Independent and dependent variable distributions of the GFJ shoppers

**Table S2**: Primary independent variable and dependent variables distributions

| **Characteristic n (%)** | **Round 1** | **Round 2** | **Round 3** |
| --- | --- | --- | --- |
| Level of GFJ exposure   - Low - Moderate - High | 34 (21.8)  25 (16)  97 (62.2) | 23 (15)  30 (19.6)  100 (65.4) | 11 (9.9)  34 (30.6)  66 (59.5) |
| Household food security   - Food secure - Food insecure (moderate & severe) | 71(45.5)  85(54.5) | 81 (52.9)  72 (47.1) | 73 (63.5)  42 (36.5) |
| General health   - Fair to poor - Good to excellent | 42 (26.9)  114 (73.1) | 42 (27.5)  111 (72.5) | 24 (20.9)  91 (79.1) |
| Mental health   - Fair to poor - Good to excellent | 17 (10.9)  139 (89.1) | 18 (11.8)  135 (88.2) | 13 (11.3)  102 (88.7) |
