# Supplementary material for: Early impact of a new food store intervention on health-related outcomes
Source: BMC Public Health. 2024 Jun 24;24:1688. doi: 10.1186/s12889-024-19052-1 (PMC11197370; doi:10.1186/s12889-024-19052-1)
Supplement: Supplementary file 2 — Supplementary Material 2 [file 12889_2024_19052_MOESM2_ESM.docx]

_Data Collector __________________________ Date _____________________ Time _____________________

**The Good Food Junction: A Community-Based Food Intervention to Address Nutritional Health Inequities**

Participant Code____________

Survey Questions:

1. Have you heard of the Good Food Junction (GFJ) Cooperative grocery store? _____Yes _____No (If no, go to question 8)

2. How did you hear about the GFJ? _____________________

3. Have you ever shopped at the Good Food Junction? ____Yes ____No (If no go to question 8) If yes, how often?

a. once _______

b. twice _______

c. three to six times _______

d. about once a month since it opened ______

e. more than once a month since it opened _______

4. What do you like best about shopping at the GFJ? Choose 1 or 2 options a. prices e. selection of food

b. location f. quality of food

c. attractiveness g. customer service

d. business model h. other: _____________

5. What do you like least about shopping at the GFJ? Choose 1 or 2 options a. prices e. selection of food

b. location f. quality of food

c. attractiveness g. customer service

d. business model h. other: _____________

6. What do you usually buy at the GFJ? Choose as many as you want.

a. fresh vegetables and fruit h. frozen vegetables or fruit b. dairy products i. pop, iced tea or energy drinks c. meat products j. hot or cold cereals d. canned food k. Other: ______________________ e. items for baking (flour, sugar, etc.)

f. snack foods (chips, pretzels, nachos, etc.)

g. pre-prepared frozen meals (lean cuisine, tv dinners, etc.)

7. Would you consider GFJ your primary grocery store? _____Yes _____No

8. What store would you consider your primary grocery store?

(make sure to include name and location)____________________________________________

9. How often do you shop for groceries? _____________________________

10. How do you usually travel to and from your primary grocery store?

___car __bus ___bike ___walk ___cab ______other

11. (If a GFJ shopper) How do you usually travel to and from the GFJ?

___car __bus ___bike ___walk ___cab ______other

12. What is the most important thing to you when choosing a grocery store? Please choose one. a. prices e. selection of food

b. location f. quality of food

c. attractiveness g. customer service

d. business model h. other: _____________

13. Do you participate in any of the following?

______ Community garden or garden allotment

______ CHEP Good Food Box

______ Food Bank or other charitable food program

______ Purchasing food directly from farmers or producers

______ Shopping at the Farmer’s Market

______ Collective kitchens

______ CHEP community markets

______ Seniors’ markets

______ Other food programs ___________________________________

14. Are you aware of any educational or promotional activities (such as food tasting, cooking demonstrations, nutrition education messages, cooking classes) hosted by the GFJ? _____Yes ______No

15. Have you participated in any of these? _____Yes ______No

16. If so, which ones?___________________________________________________________ 17. Would you be willing to be contacted to participate in further research? _____Yes ______No

_Data Collector __________________________ Date _____________________ Time _____________________

**Demographic Questions**

Participant Code____________

Name ______________________ Age______________________ Sex ____F _______M Occupation______________________

Address _________________________ Postal Code___________________________

Do you identify as:

Aboriginal ______Yes ____No

Senior (65+) _____Yes _____No

New Immigrant (less than 5 years in Canada) _____Yes _____No

Including yourself, how many people live in this house? 1 2 3 4 5 6+

Who lives with you? Check as many as apply ____ My partner or spouse

____ My children

____ Other relatives (parents, grandparents, aunts, uncles, cousins, nieces,

nephews)

____ Roommates

____ Foster children

____ Other

If children live in your house, how many are under the age of 18? ___________

What is the highest level of education that you have completed?

___ less than high school

___ completed high school

___ some college (SIAST)

___ completed college (SIAST)

___ some university

___ completed university

___ declined to answer

What is your marital status?

____ Married or living common law

____ Divorced

____ Separated

____ Widowed

____ Single - never married

Now I am going to ask you about your household annual income. Please stop me when I have read the category which applies to your household. Was it...?

____ Less than $5,000

____ $5,000 to less than $10,000

____$10,000 to less than $15,000

____$15,000 to less than $20,000

____$20,000 to less than $30,000

____$30,000 to less than $40,000

____$40,000 to less than $50,000

____$50,000 to less than less than $60,000

____$60,000 to less than less than $70,000

____$70,000 to less than less than $80,000

____$80,000 to less than less than $90,000

____$90,000 to less than less than $100,000

____$100,000 to less than less than $150,000

____$150,000 and over

Date__________________ Data Collector___________________ Participant Code___________________

**Good Food Junction Research Health Status Questionnaire**

1. In general, would you say your health is... ?

a. Excellent

b. Very good

c. Good

d. Fair

e. Poor

2. Compared to one year ago, how would you say your health is now? Is it...?

a. Much better now than 1 year ago

b. Somewhat better now (than 1 year ago)

c. About the same as 1 year ago

d. Somewhat worse now (than 1 year ago)

e. Much worse now (than 1 year ago)

3. In general, would you say your mental health is...?

a. Excellent

b. Very good

c. Good

d. Fair

e. Poor

4. Thinking about the amount of stress in your life, would you say that most days are...?

a. Not at all stressful

b. Not very stressful

c. A bit stressful

d. Quite a bit stressful

e. Extremely stressful

5. In general, what kind of neighbourhood would you say you live in? Would you say it is a neighbourhood in which people do things together and try to help each other, or one in which people mostly go their own way?

a. Help each other out

b. Go their own way

c. Mixture

d. Refused

e. N/A

1

Date__________________ Data Collector___________________ Participant Code___________________

6. How much do you feel a part of your neighborhood?

a. Very much a part

b. Somewhat a part

c. Not very much a part d. Refused

e. N/A

7. If there was a neighbourhood project organized, such as a block party or yard sale, how comfortable would you feel about participating?

a. Very comfortable

b. Somewhat comfortable

c. Uncomfortable

d. Refused

e. N/A

8. Do you feel comfortable calling upon your neighbours for assistance or help during a crisis?

a. Yes

b. No

c. Refused

d. N/A

9. In the past 12 months, did you do anything to improve your health? (For example, lost weight, quit smoking, increased exercise)

a. Yes

b. No

10. What is the single most important change you have made?

a. Increased exercise, sports / physical activity

b. Lost weight

c. Changed diet / improved eating habits

d. Quit smoking / reduced amount smoked

e. Drank less alcohol

f. Reduced stress level

g. Received medical treatment

h. Took vitamins

i. Other

2

Date__________________ Data Collector___________________ Participant Code___________________

11. Do you think there is [anything else/anything] you should do to improve your physical health?

a. Yes

b. No

12. What is the most important thing?

a. Start / Increase exercise, sports / physical activity

b. Lose weight

c. Change diet / improve eating habits

d. Quit smoking / reduce amount smoked

e. Drink less alcohol

f. Reduce stress level

g. Receive medical treatment

h. Take vitamins

i. Other

13. Is there anything stopping you from making this improvement?

a. Yes

b. No

14. What is that?

a. Lack of will power / self-discipline

b. Family responsibilities

c. Work schedule

d. Addiction to drugs / alcohol

e. Physical condition

f. Disability / health problem

g. Too stressed

h. Too costly / financial constraints

i. Not available - in area

j. Transportation problems

k. Weather problems

l. Other

15. Is there anything you intend to do to improve your physical health in the next year?

a. Yes

b. No

3

Date__________________ Data Collector___________________ Participant Code___________________

16. What is that?

a. Start / Increase exercise, sports / physical activity

b. Lose weight

c. Change diet / improve eating habits

d. Quit smoking / reduce amount smoked

e. Drink less alcohol

f. Reduce stress level

g. Receive medical treatment

h. Take vitamins

i. Other

14. Have you ever been told by a medical provider that you have: Diabetes?

a. Yes

b. No

High blood pressure?

a. Yes

b. No

Heart disease?

a. Yes

b. No

Cancer?

a. Yes

b. No

What is your height?______________________

What is your weight?______________________

The next questions are about the foods you usually eat or drink. Think about all the foods you eat, both meals and snacks, at home and away from home.

17. How often do you usually drink fruit juices such as orange, grapefruit or tomato? (For example: once a day, three times a week, twice a month) a. ________ Per day

b. ________ Per week

c. _______ Per month

d. ________ Per year

e. Never

4

Date__________________ Data Collector___________________ Participant Code___________________

18. Not counting juice, how often do you usually eat fruit?

a. ________ Per day

b. ________ Per week

c. _______ Per month

d. ________ Per year

e. Never

19. How often do you (usually) eat green salad?

a. ________Per day

b. ________ Per week

c. _______ Per month

d. ________ Per year

e. Never

20. How often do you usually eat potatoes, not including French fries, fried potatoes, or potato chips?

a.________ Per day

b. ________ Per week

c. _______ Per month

d. ________ Per year

e. Never

21. How often do you (usually) eat carrots?

a.________ Per day

b. ________ Per week

c. _______ Per month

d. ________ Per year

e. Never

22. Not counting carrots, potatoes, or salad, how many servings of other vegetables do you usually eat?

a. ________ Per day

b. ________ Per week

c. _______ Per month

d. ________ Per year

e. Never

The following questions are about the food situation for your household in the past 12 months.

23. Which of the following statements best describes the food eaten in your household in the past 12 months, that is, since ^CURRENTMONTH of last year?

5

Date__________________ Data Collector___________________ Participant Code___________________

a. You and Others always had enough of the kinds of food you wanted to eat.

b. You and Others had enough to eat, but not always the kinds of food you wanted.

c. Sometimes You and Others did not have enough to eat.

d. Often You and Others didn’t have enough to eat

Now I’m going to read you several statements that may be used to describe the food situation for a household. Please tell me if the statement was often true, sometimes true, or never true for You and Others in the past 12 months.

The first statement is:

24. You and Others worried that food would run out before you got money to buy more. Was that often true, sometimes true, or never true in the past 12 months?

a. Often true

b. Sometimes true

c. Never true

25. The food that You and Others bought just didn’t last, and there wasn’t any money to get more. Was that often true, sometimes true, or never true in the past 12 months?

a. Often true

b. Sometimes true

c. Never true

26. You and Others couldn’t afford to eat balanced meals. In the past 12 months was that often true, sometimes true, or never true?

a. Often true

b. Sometimes true

c. Never true

Now I’m going to read a few statements that may describe the food situation for households with children.

27. You and other adults relied on only a few kinds of low-cost food to feed your child because you were running out of money to buy ood. Was that often true, sometimes true, or never true in the past 12 months?

a. Often true

b. Sometimes true

c. Never true

6

Date__________________ Data Collector___________________ Participant Code___________________

28. You and other adults couldn’t feed your child a balanced meal, because you couldn’t afford it. Was that often true, sometimes true, or never true in the past 12 months?

a. Often true

b. Sometimes true

c. Never true

29. Your child not eating enough because you and other adults just couldn't afford enough food. Was that often, sometimes, or never true in the past 12 months?

a. Often true

b. Sometimes true

c. Never true

The following few questions are about the food situation in the past 12 months for you or any other adults in your household.

30. In the past 12 months, since last ^CURRENTMONTH did You and other adults ever cut the size of your meals or skip meals because there wasn’t enough money for food?

a. Yes

b. No

31. How often did this happen---almost every month, some months but not every month, or in only 1 or 2 months?

a. Almost every month

b. Some months but not every month

c. Only 1 or 2 months

32. In the past 12 months, did you (personally) ever eat less than you felt you should because there wasn't enough money to buy food?

a. Yes

b. No

33. In the past 12 months, were you (personally) ever hungry but didn't eat because you couldn't afford enough food?

a. Yes

b. No

7

Date__________________ Data Collector___________________ Participant Code___________________

34. In the past 12 months, did you (personally) ever lose weight because you didn't have enough money for food?

a. Yes

b. No

35. In the past 12 months, did you or other adults ever not eat for a whole day because there wasn't enough money for food?

a. Yes

b. No

36. How often did this happen...? Almost every month, some months but not every month, or in only 1 or 2 months?

a. Almost every month

b. Some months but not every month

c. Only 1 or 2 months

Now, a few questions on the food experiences for children in your household.

37. In the past 12 months, did you or other adults ever cut the size of any child’s meals because there wasn't enough money for food?

a. Yes

b. No

38. In the past 12 months, did any child ever skip meals because there wasn't enough money for food?

a. Yes

b. No

39. How often did this happen...? Almost every month, some months but not every month, or in only 1 or 2 months?

a. Almost every month

b. Some months but not every month

c. Only 1 or 2 months

40. In the past 12 months, was any child ever hungry but you just couldn't afford more food?

a. Yes

b. No

8

Date__________________ Data Collector___________________ Participant Code___________________

41. In the past 12 months, did any child ever not eat for a whole day because there wasn't enough money for food?

a. Yes

b. No

9
